# The effect of high-polyphenol Mediterranean diet on visceral adiposity: the DIRECT PLUS randomized controlled trial

**DOI:** 10.1186/s12916-022-02525-8

**Published:** 2022-09-30

**Authors:** Hila Zelicha, Nora Kloting, Alon Kaplan, Anat Yaskolka Meir, Ehud Rinott, Gal Tsaban, Yoash Chassidim, Matthias Bluher, Uta Ceglarek, Berend Isermann, Michael Stumvoll, Rita Nana Quayson, Martin von Bergen, Beatrice Engelmann, Ulrike E. Rolle-Kampczyk, Sven-Bastiaan Haange, Kieran M. Tuohy, Camilla Diotallevi, Ilan Shelef, Frank B. Hu, Meir J. Stampfer, Iris Shai

**Affiliations:** 1 Faculty of Health Sciences, The Health & Nutrition Innovative International Research Center, Ben-Gurion University of the Negev, P.O. Box 653, 84105 Be’er Sheva, Israel; 2 Department of Medicine, University of Leipzig, Leipzig, Germany; 3 Department of Engineering, Sapir Academic College, Ashkelon, Israel; 4 Helmholtz Institute for Metabolic, Obesity and Vascular Research (HI-MAG) of the Helmholtz Zentrum München at the University of Leipzig and University Hospital Leipzig, Leipzig, Germany; 5 Department of Food Quality and Nutrition, Research and Innovation Centre, Fondazione Edmund Mach, San Michele all’Adige, Trentino, Italy; 6 Soroka University Medical Center, Be’er Sheva, Israel; 7 Department of Epidemiology, Harvard T.H. Chan School of Public Health, Boston, MA USA; 8 Harvard Channing Division of Network Medicine, Department of Medicine, Brigham and Women’s Hospital, Boston, MA USA; 9 Department of Nutrition, Harvard T.H. Chan School of Public Health, Boston, MA USA

**Keywords:** Mediterranean, Obesity, Plant-based diet, Polyphenols, Visceral adipose tissue

## Abstract

**Background:**

Mediterranean (MED) diet is a rich source of polyphenols, which benefit adiposity by several mechanisms. We explored the effect of the green-MED diet, twice fortified in dietary polyphenols and lower in red/processed meat, on visceral adipose tissue (VAT).

**Methods:**

In the 18-month Dietary Intervention Randomized Controlled Trial PoLyphenols UnproceSsed (DIRECT-PLUS) weight-loss trial, 294 participants were randomized to (A) healthy dietary guidelines (HDG), (B) MED, or (C) green-MED diets, all combined with physical activity. Both isocaloric MED groups consumed 28 g/day of walnuts (+ 440 mg/day polyphenols). The green-MED group further consumed green tea (3–4 cups/day) and *Wolffia globosa* (duckweed strain) plant green shake (100 g frozen cubes/day) (+ 800mg/day polyphenols) and reduced red meat intake. We used magnetic resonance imaging (MRI) to quantify the abdominal adipose tissues.

**Results:**

Participants (age = 51 years; 88% men; body mass index = 31.2 kg/m^2^; 29% VAT) had an 89.8% retention rate and 79.3% completed eligible MRIs. While both MED diets reached similar moderate weight (MED: − 2.7%, green-MED: − 3.9%) and waist circumference (MED: − 4.7%, green-MED: − 5.7%) loss, the green-MED dieters doubled the VAT loss (HDG: − 4.2%, MED: − 6.0%, green-MED: − 14.1%; *p* < 0.05, independent of age, sex, waist circumference, or weight loss). Higher dietary consumption of green tea, walnuts, and *Wolffia globosa*; lower red meat intake; higher total plasma polyphenols (mainly *hippuric acid*), and elevated urine *urolithin A* polyphenol were significantly related to greater VAT loss (*p* < 0.05, multivariate models).

**Conclusions:**

A green-MED diet, enriched with plant-based polyphenols and lower in red/processed meat, may be a potent intervention to promote visceral adiposity regression.

**Trial registration:**

ClinicalTrials.gov, NCT03020186

**Supplementary Information:**

The online version contains supplementary material available at 10.1186/s12916-022-02525-8.

## Background

Visceral adipose tissue (VAT) accumulation is one of the main key factors that differentiate between metabolic healthy and unhealthy obese individuals [1, 2]. VAT is closely related to the development of multiple cardiovascular risk factors, including hypertension, dyslipidemia, type 2 diabetes (T2D), and an independent marker of mortality [3–7]. Moreover, VAT was found independently associated with elevated 10-year cardiovascular risk, particularly in men, and is suggested as a tool for long-term cardiovascular disease (CVD) risk assessment [8]. In contrast, subcutaneous adipose tissue (SAT) is inconsistently associated with obesity-related morbidity [9–11]. While superficial SAT is correlated with improved glycemic control and indices of cardiovascular health [10], deep SAT is correlated with high blood pressure, obesity, and insulin resistance [9].

The Mediterranean (MED) diet, high in polyphenol content [12] and rich in plant food sources, was shown to have an enhanced effect on VAT reduction in combination with physical activity (PA), regardless of weight loss [13]. Polyphenols are diverse phytochemicals, common in plant-based foods, widely studied in recent years due to their possible antioxidant and anti-inflammatory properties and the potential for preventing unhealthy metabolic obesity, T2D, CVD, and hypertension [2, 14, 15]. As for the effect of polyphenols on adiposity, various mechanisms have been proposed, mostly based on animal and cell studies, including inhibition of adipocytes differentiation, increased fatty acid oxidation, decreased fatty acid synthesis, increased thermogenesis, and energy expenditure [16–22].

In our previous randomized controlled trials (e.g., DIRECT [23, 24], CENTRAL [13], CASCADE [25]), dietary interventions richer in polyphenol content tended to yield more successful cardiometabolic results, as well as mobilization of specific ectopic fat depots. Thus, we aimed to assess the effect of the MED diet, further enriched with polyphenols (green tea and *Wolffia globose* Mankai plant, high-quality green plant-based protein-rich in polyphenols), and lower in red and processed meat (“green-MED diet”) on visceral adiposity in the 18-month Dietary Intervention Randomized Controlled Trial-Polyphenols, Unprocessed (DIRECT-PLUS) trial.

## Results

### Baseline characteristics

DIRECT-PLUS participants (Table 1; Additional file 1: Fig. S1) were 50.8 (10.4) years of age on average, and mostly men (88%), commensurate with the workplace, with abdominal obesity [mean WC = 110 cm (men) and 103 cm (women)], mean BMI = 31.2 (3.9) kg/m^2^, 36.3% participants with pre-diabetes, and 11.3% participants with type 2 diabetes. The mean areas of abdominal adipose tissues and blood biomarkers were similar across the intervention groups (Table 1). Baseline correlations between the three abdominal adipose depots, clinical parameters, and cardiovascular risk score are reported in Additional file 1: Fig. S2. The VAT area at baseline was more strongly correlated with WC (*r* = 0.54, *p* < 0.001) than with bodyweight (*r* = 0.38, *p* < 0.001). Greater VAT was associated with higher cardiovascular risk score (*r* = 0.41), systolic (*r* = 0.36) and diastolic blood pressure (*r* = 0.28), triglycerides (*r* = 0.21), glucose (*r* = 0.30), HOMA-IR (*r* = 0.47), and IL-6 (*r* = 0.30) and lower HDL-c levels (*r* = − 0.18). Deep SAT accumulation was associated with higher HOMA-IR (*r* = 0.24), weight (*r* = 0.60), WC (*r* = 0.63), cardiovascular risk score (*r* = 0.20), and IL-6 (*r* = 14). In contrast, greater superficial SAT was associated with lower cardiovascular risk score (*r* = − 0.36), systolic blood pressure (*r* = − 0.13), and triglycerides (*r* = − 0.21).

**Table 1:** Baseline characteristics of the DIRECT-PLUS study population* (*n* = 286)

	HDG (***n*** = 98)	MED (***n*** = 96)	Green-MED (***n*** = 92)	Entire (***n*** = 286)
Men, % of the study population	86 (87.8%)	85 (88.5%)	81 (88.0%)	252 (88.1%)
Age, years	51.1 (10.6)	51.5 (10.5)	49.8 (10.1)	50.8 (10.4)
BMI, kg/m^2^	31.2 (3.8)	31.2 (4.0)	31.2 (4.0)	31.2 (3.9)
WC, cm				
Men	110.7 (10.1)	110.7 (9.4)	110.0 (7.3)	110.5 (9.0)
Women	103.8 (9.7)	104.9 (10.0)	100.8 (9.9)	103.2 (9.7)
Blood pressure, mmHg				
Diastolic	80.2 (11.3)	81.8 (8.9)	81.0 (10.0)	81.0 (10.1)
Systolic	130.2 (14.3)	130.1 (12.5)	129.8 (15.1)	130.1 (13.9)
*Blood biomarkers*				
Ratio of triglycerides to HDL cholesterol	3.8 (2.5)	3.6 (2.1)	3.4 (2.0)	3.6 (2.2)
Serum LDL cholesterol, mg/dL	126.8 (32.3)	127.1 (31.3)	124.0 (28.6)	126.0 (30.7)
Ratio of LDL to HDL cholesterol	2.9 (0.9)	2.9 (1.0)	2.9 (1.0)	2.9 (1.0)
Serum HDL cholesterol, mg/dL				
Men	43.4 (9.9)	46.0 (10.0)	43.0 (10.7)	44.2 (10.2)
Women	59.6 (12.6)	53.5 (16.2)	62.0 (13.4)	58.4 (14.1)
Fasting glucose, mg/dL	102.1 (17.7)	100.6 (13.4)	102.9 (20.0)	101.8 (17.2)
Fasting insulin, μIU/mL	15.3 (9.0)	14.4 (7.1)	14.1 (7.3	14.6 (7.9)
HOMA-IR	4.0 (2.8)	3.6 (1.9)	3.6 (2.2)	3.7 (2.3)
HbA_1C_				
%	5.5 (0.7)	5.4 (0.5)	5.5 (0.7)	5.5 (0.7)
mmol/mol	37 (7.3)	36 (4.3)	37 (7.3)	37 (7.3)
C-reactive protein, mg/L	3.0 (2.1)	3.1 (1.8)	3.0 (2.1)	3.0 (2.0)
Chemerin, ng/mL	205.9 (44.1)	208.6 (43.4)	207.7 (42.7)	207.4 (43.3)
Leptin, ng/mL	13.7 (12.2)	15.0 (12.4)	14.3 (11.9)	14.3 (12.1)
*Abdominal adipose tissue area, cm*^*2*^				
Visceral adipose tissue, cm^2^	134.3 (49.3)	130.5 (43.6)	130.4 (53.7)	131.8 (48.8)
Deep subcutaneous adipose tissue, cm^2^	218.5 (73.1)	217.6 (62.8)	212.6 (64.7)	216.3 (66.9)
Superficial subcutaneous adipose tissue, cm^2^	115.3 (45.9)	117.1 (45.5)	114.3 (55.3)	115.6 (49.0)
*Proportion, %*				
Visceral adipose tissue				
Men	30.3 (8.6)	28.6 (7.4)	30.7 (9.6)	29.9 (8.6)
Women	19.6 (3.1)	24.4 (7.3)	16.6 (6.8)^†^	19.9 (6.5)
Deep subcutaneous adipose tissue				
Men	46.5 (6.1)	47.6 (5.8)	46.7 (6.7)	46.9 (6.2)
Women	45.3 (3.2)	39.6 (8.9)	43.3 (5.8)	43.0 (6.3)
Superficial subcutaneous adipose tissue				
Men	23.2 (5.4)	23.8 (5.4)	22.6 (5.7)	23.2 (5.5)
Women	35.1 (3.5)	35.9 (6.0)	40.1 (8.5)	37.0 (6.5)

Significance between the groups was assessed according to the ANOVA/Kruskal-Wallis test for continuous variables and chi-square for categorical variables

*BMI* body mass index, *HDG* healthy dietary guidelines, *HDL-c* high-density lipoprotein cholesterol, *HOMA-IR* homeostatic model of insulin resistance, *LDL-c* low-density lipoprotein cholesterol, *MED* Mediterranean, *WC* waist circumference

*Values are presented as mean (standard deviations) for continuous variables and as number and % for categorical variables. A total of 286 available abdominal adipose tissue MRI

^†^The visceral adipose tissue proportion among the women population was significant across the groups (*p* = 0.02) between the green-MED and the MED groups

### Eighteen-month change in abdominal adipose tissues, weight, and waist circumference

The DIRECT-PLUS retention rate was 98.3% after 6 months and 89.8% after 18 months; 79.3% had eligible follow-up MRIs. Attribution was due to the lack of motivation and medical reasons unrelated to the study. The 18-month dropout rate did not significantly differ across the intervention groups (*p* = 0.28). As previously reported [26, 27], the participants assigned to the green-MED diet consumed more green tea and, exclusively, Mankai green shake and reduced their red meat and poultry intake compared to those assigned to the MED diet (*p* < 0.05 for all comparisons between the MED groups), and all three intervention groups similarly increased their PA levels, measured in MET units. Additional adherence information is presented in S1.

The mean weight loss (HDG: − 0.4% (5.0), MED: − 2.7% (5.6), green-MED: − 3.9% (6.5)) and WC loss (HDG: − 3.6% (5.1), MED: − 4.7% (5.0), green-MED: − 5.7%(5.7)) after 18 months were similar between the two MED diets (*p* > 0.05 for all) and higher as compared to the HDG (weight: HDG vs. MED: *p* = 0.02; HDG vs. green+MED: *p* < 0.001; WC: HDG vs. MED: *p* = 0.33, HDG vs. green+MED: *p* = 0.02).

**Fig. 1:**
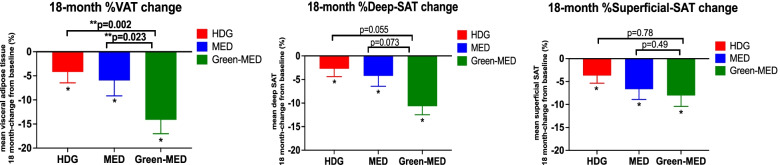
Eighteen-month changes in abdominal adipose tissues (mean (SE)) between the intervention groups (*n* = 286). After 18 months of intervention, all groups reduced all three abdominal adipose tissues significantly. Significant differences in VAT% change between the green-MED group and MED, as well as HDG groups, were observed after adjustment for age, sex, and waist circumference change. Deep SAT, deep subcutaneous; superficial SAT, superficial subcutaneous; HDG, healthy dietary guidelines; MED, Mediterranean; VAT, visceral adipose tissue. *Significant within-group change vs. baseline at the 0.05 level. **Significant differences between the groups at the 0.05 level

**Fig. 2:**
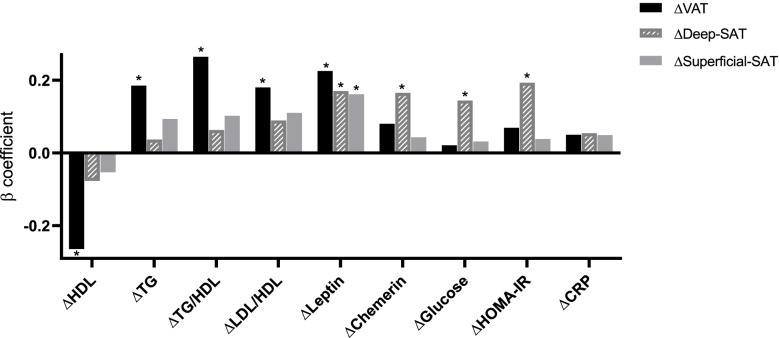
Multivariate linear regression models were adjusted for age, sex, weight changes, and the three intervention groups. We aimed to identify the independent associations between the changes in abdominal fat depots and biomarkers in multivariate models, adjusted for age, sex, intervention group, and weight loss. While VAT loss was independently associated with an improved lipid profile, the deep SAT loss was independently and significantly associated with beneficial glycemic biomarkers during the intervention (*p* < 0.05 for all). The association between changes in blood biomarkers and reduction in abdominal fat subdepots is presented by the *β* standardized coefficient. *p < 0.05 considered statistically significant. HDL-c, high-density lipoprotein cholesterol; HOMA-IR, homeostatic model of insulin resistance; LDL-c, low-density lipoprotein cholesterol

All three abdominal fat depots decreased over 18 months of intervention (*p* < 0.05 vs. baseline for all). The green-MED group achieved a greater reduction in VAT than the other intervention groups (HDG: − 4.2% (22.5), MED: − 6.0%(31.3), green-MED: − 14.1%(27.7); *p* < 0.05 green-MED vs. MED or vs. HDG groups). These differences in VAT loss across the groups remained significant after adjusting for age, sex, and 18-month WC change (green-MED vs. MED *p* = 0.023; green-MED vs. HDG *p* = 0.002) (Fig. 1). After adjustment for weight change, differences between the MED groups remained significant (*p* = 0.042), but the difference between the HDG and green-MED groups was attenuated (*p* = 0.07). Sensitivity analysis among men only (the majority of this cohort) is presented in S2 and Additional file 1: Fig. S3. Changes in superficial SAT and deep SAT were not statistically different between the intervention groups after adjustment for WC or weight change. Illustrative MRI images of three participants with similar baseline characteristics and WC change show greater VAT loss in the green-MED group than in other intervention groups (Additional file 1: Fig. S4). Associations between changes in abdominal adipose depots and cardiometabolic biomarkers adjusted for age, sex, weight changes, and the three intervention groups are reported in Fig. 2. While VAT loss was independently associated with an improved lipid profile, the deep SAT loss was independently and significantly related to beneficial glycemic biomarkers during the intervention (*p* < 0.05 for all).

### Specific dietary components and VAT loss

**Fig. 3:**
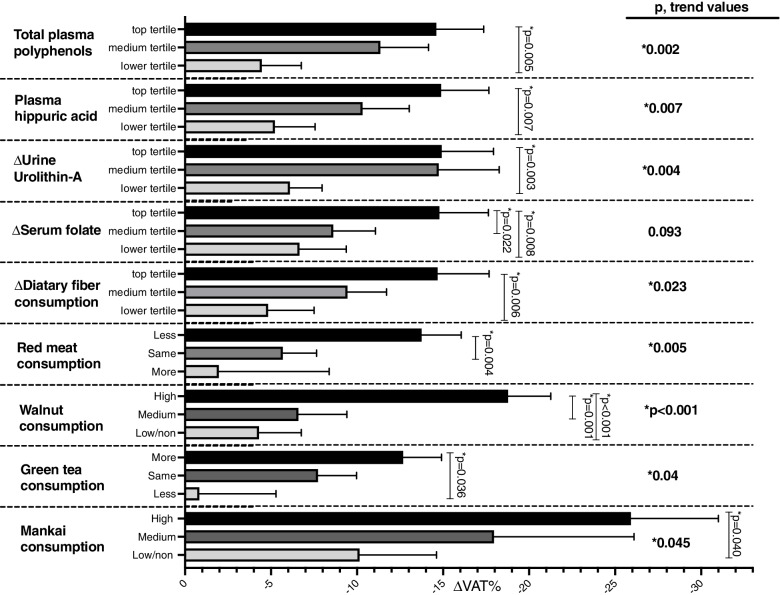
Multivariate models for the assessment of the associations between nutritional components of the green-MED diet with changes in VAT% adjusted for age and sex. Mankai consumption was adjusted for age and referred to the green-MED group only. Mankai consumption categories (18 months): low/non: ≤ 1/week, medium: 2–3/week, and high: > 3/week; walnut consumption categories (18 months): low/non: 0 to 1–3 times/month, medium: 1–2/week to 3–4/week, and high: more than 5–6/week; serum folate tertiles (of 18-month change in serum folate, ng/dL): lower ≤ − 0.41, medium − 0.40 to 1.46, and top ≥ 1.47; fiber consumption tertiles (18-month change, g): lower ≤ − 6.73, medium − 6.72 to − 0.17, and top ≥ − 0.16; plasma polyphenol tertiles (18 months, mg/L): lower ≤ 0.23, medium 0.24 to 0.47, and top ≥ 0.48; specific polyphenols (urine and plasma) and VAT change after 18 months of intervention adjusted for age and sex: urine *urolithin-A* delta 18 months compared to baseline (log2) tertiles: T1 ≤ 0, T2 = 0 to 4.92, and T3 = 4.92+. *r* = − 0.241, *p* < 0.001, *q* = 0.00036 (MC -139 metabolites). Plasma *Hippuric-acid* tertiles (time18, mg/L): T1 ≤ 0.21, T2 = 0.21 to 0.44, and T3 = 0.44+. *Significant differences between the groups at the 0.05 level. VAT, visceral adipose tissue

Higher dietary consumption of green tea, walnuts, and dietary fiber and reduced red meat consumption were significantly associated with greater %VAT loss (age- and sex-adjusted, *p* < 0.05 for all) (Fig. 3). Walnut consumption, reduced red meat consumption, and increased dietary fiber consumption remained significantly associated with VAT loss after further adjustment for WC loss (*p* < 0.05 for all). The association of green tea intake with VAT loss was attenuated after adjustment for WC change. Furthermore, after adjusting for weight loss, increased dietary fiber consumption remained significantly associated with VAT loss (*p* < 0.001), but all other dietary components were attenuated. Within the green-MED group (the only group with *Wolffia globosa* (Mankai)), increased intake of Mankai was significantly associated with greater VAT reduction (with a 26% VAT loss at the highest intake level (≥ 3/week); *p* = 0.04 vs. the lowest level, age-adjusted] (Fig. 3). Higher Mankai consumption was also associated with a reduction in CVD risk and improved lipid profile (total cholesterol: *p* = 0.023; TG/HDL: *p* = 0.044; SCORE: *p* = 0.038; adjusted for age and weight-loss) (Additional file 1: Fig. S5).

We observed a significant synergistic interaction effect between decreased red meat consumption and increased serum folate on VAT loss (*p*, interaction = 0.048, age- and sex-adjusted) (Additional file 1: Fig. S6). VAT loss was significantly higher (− 21.7%) among participants who both reduced red meat consumption and increased serum folate (top tertile) compared to those whose red meat consumption and serum folate remained unchanged (− 5.5%; *p* = 0.013 between the groups).

As previously reported [28], after 18 months, the total plasma polyphenol levels were higher in both MED groups (0.47 (0.4) mg/L for both) as compared to the HDG group (0.35 (0.4) mg/L; *p* < 0.05 for both MED vs. HDG).

Higher levels of total plasma polyphenol and serum folate [29] may reflect higher consumption of “green” dietary components, which were significantly associated with greater VAT loss (age- and sex-adjusted, *p* < 0.05 for all) (Fig. 3). After further adjusting for WC loss, total plasma polyphenol levels remained significantly associated with VAT loss (*p* = 0.018). Specifically, among plasma polyphenols examined, *hippuric acid* levels at 18 months were significantly higher in both MED diets than in the HDG group (*p* < 0.05) and were associated with VAT loss in a multivariate model adjusted for age and sex (Fig. 3) and were still significant after additional adjustment for WC change (*p* = 0.015, low vs. high tertile).

An exploratory analysis of urine polyphenol compounds showed an increase in *urolithin A* was strongly correlated with reduced VAT, even after adjusting for multiple comparisons for 139 identified metabolites (*r* = − 0.241, *p* < 0.001, *q* = 0.00036). *Urolithin A* was significantly associated with lower VAT (Fig. 3; adjusting for age and sex) and remained significant after adjusting for WC change. The increase in *urolithin A* was significantly correlated with increased consumption of walnuts (*r* = 0.14, *p* = 0.035) and Mankai (*r* = 0.24, *p* = 0.044).

## Discussion

In this 18-month dietary intervention study, the green-MED diet, richer in dietary polyphenols and green plant-based proteins and lower in red meat, might be a more effective strategy for VAT loss than the traditional healthy MED diet achieving more than twice the degree of VAT reduction, despite similar weight loss. VAT loss was specifically related to lower red meat intake and increased walnuts, green tea, *Wolffia globosa*, and dietary fiber and was reflected by higher plasma polyphenol and serum folate levels*.* We identified specific polyphenols whose elevation predicted greater VAT loss. This study may suggest an improved dietary protocol for treating visceral adiposity.

Several limitations should be acknowledged. The low proportion of women reflects the workplace, and different VAT proportions at baseline across groups limit the generalizability of findings to women nor can we not identify the exact components responsible for the dietary effects as we compared dietary regimens and not specific nutrients. We assessed adherence by the self-reported dietary intake assessment tool, which is subject to error, although the instrument has been validated [30]. Yet, we analyzed the serum folate levels, which can reflect green leaf consumption [29]. Total lean body mass or fat mass measurements were not available from our MRI analysis. Abdominal adipose tissues were measured in a semiautomatic manner and recorded as area and not volume. However, we observed high inter- and intraclass correlations, supporting their reproducibility. The recommended PA was monitored by self-report for all groups and not direct objective means. The dietary assessment was inadequate to estimate the intake of polyphenols beyond the evaluation of the specific high polyphenol foods provided. Plasma and urinary polyphenol assessments provide objective data; however, these measurements are limited in reflecting polyphenol intake. Additionally, the urine polyphenol analysis was based on a spot sample rather than a 24-h collection. In general, we tried to confirm the beneficial effects of dietary polyphenols in a dietary pattern human study, as suggested in lab-based experiments. The strengths of the study include the relatively large sample size, high retention rate, and use of 3-T MRI measurements (considered one of the gold standards tools for the quantification of specific fat depots [31]) and the division of SAT into deep and superficial fat tissues, which are known to differ histologically and physiologically [32, 33]. Furthermore, the closed workplace enabled monitoring of the freely provided lunch, the presence of an onsite clinic, intense dietary guidance and group meetings with multidisciplinary guidance, and access to polyphenol-rich foods provided at no charge.

Our findings further support the clinical significance of different abdominal fat depots. In both the CENTRAL [13] and DIRECT-PLUS trials, after diet-induced weight loss, VAT reduction was associated with an improved lipid profile, as the deep SAT reduction was associated with a beneficial glycemic profile. The superficial SAT was previously correlated with improved glycemic control (HbA1c and fasting glucose) and better indicators of cardiovascular health [10]. These differences may be explained by differential sensitivity to lipolytic stimulation hormones. VAT adipocytes show higher lipogenic and lipolytic activity and produce more proinflammatory cytokines, while subcutaneous adipocytes are the main source of leptin [34]. Therefore, a reduction in VAT accumulation, known as a key risk factor in CVD development, may reduce metabolic complications, improve the lipid profile, and decrease cardiometabolic risk. The two subcutaneous depots differ histologically and physiologically, with deep SAT having higher lipolytic activity and larger, polygonal, and better-organized fat lobules than the superficial SAT depot [32, 33]. This study further reinforces the hypothesis that the distribution of abdominal subdepots may be a key factor in cardiometabolic risk rather than total body weight.

The positive health effects of the traditional MED diet, moderately high in PUFAs and MUFAs and low in red meat, are well-established and recognized [13, 23, 35–38]. However, a randomized crossover trial showed that the low-fat vegan diet successfully induced weight reduction and lipid profile compared to the MED diet for 16 weeks [39]. As other studies did not find a significant difference between a low-fat diet and a MED diet in VAT reduction [40, 41], the specific dietary components that may affect body fat distribution are uncertain. In some studies, different dietary patterns exhibited no differential effect on specific abdominal fat depots [42, 43], whereas others suggest that intake of simple carbohydrates [44] and red/processed meats [45] specifically increase VAT; fruit and whole-grain intake [45], MUFA, and PUFA decrease VAT accumulation [46]. Recently published as part of the DIRECT-PLUS, the prevalence of nonalcoholic fatty liver disease was reduced by half by the strategy of the green Mediterranean diet [28]. The current analyses showed that the green-MED diet could improve the traditional MED diet for VAT reduction. In both analyses, greater IHF [28] and VAT loss were independently associated with increased *Wolffia globosa* and walnut intake, decreased red meat consumption, and improved serum folate. However, VAT loss was also associated with a higher intake of green tea and dietary fiber. *Wolffia globosa*, which had the highest magnitude of VAT reduction, is an aquatic plant rich in polyphenols [47] and high-quality protein [48] with beneficial effects on postprandial and fasting glycemic control [49], known to provide bioavailable essential amino acids, iron, and available B12 vitamin.

The beneficial effects of the green-MED diet on VAT loss might be explained by polyphenols. In the DIRECT-PLUS, we noted a significant association between total plasma polyphenols and VAT loss that remained significant after adjusting for WC change. While exploring specific polyphenols, we found that *urolithin A*, a derived gut microbiota metabolite of ellagitannin, was significantly correlated with VAT loss. In a recent mouse experiment, *urolithin A* was found to be an anti-obesity agent, increasing energy expenditure by enhancing thermogenesis in brown adipose tissue and inducing browning of white adipose tissue [17]. In our trial, elevated *urolithin A* was correlated with walnut and Mankai consumption, as its precursor, ellagic acid [50], was found in both. In addition, plasma *hippuric acid*, a glycine conjugate of benzoic acid, both metabolites present in Mankai, was significantly associated with VAT reduction even after controlling for WC change. *Hippuric acid* is an end product of microbial metabolism of different classes of dietary polyphenols, and elevated fasting plasma levels indicate an upregulation of microbiome total polyphenol metabolism. *Hippurate* appeared to be the single most important metabolite linking diet and visceral fat [51].

## Conclusion

A green-MED diet enriched with polyphenols and decreased red meat consumption might serve as an improved version of the MED diet for targeted VAT reduction. Future studies are needed to explore the exact mechanisms of specific polyphenol-rich foods on visceral adiposity.

## Methods

### Study design

The DIRECT-PLUS trial (ClinicalTrials.gov NCT03020186), initiated in May 2017, was conducted in an isolated workplace (Negev Nuclear Research Center, Dimona, Israel), where a monitored lunch was provided. Of the 378 volunteers, 294 met the inclusion criteria: 30+ years of age with abdominal obesity [waist circumference (WC): men > 102 cm, women > 88 cm] or dyslipidemia [triglycerides > 150 mg/dL and high-density lipoprotein cholesterol (HDL-c): men ≤ 40 mg/dL, women: ≤ 50 mg/dL]. The exclusion criteria are fully described in S3. The Soroka University Medical Centre Medical Ethics Board and the Institutional Review Board approved the study protocol for the DIRECT PLUS trial. All participants provided written informed consent and received no financial compensation.

### Randomization and intervention

Participants who completed the baseline measurements were randomly assigned to one of three intervention groups (1:1:1 ratio), stratified by sex and working sites: healthy dietary guidelines (HDG), MED diet, or green-MED diet, all included PA recommendations, with a free gym membership and educational sessions promoting moderate-intensity PA [13] with ~ 80% aerobic content (S4). Dietary and PA interventions are fully described in Additional file 1: Table S1. Randomization was conducted in a single phase, as the interventions were conducted simultaneously, and participants were aware of their assigned intervention (open-label protocol). The HDG group received basic health promotion guidelines to achieve a healthy diet. The MED group was instructed to follow a calorie-restricted traditional MED diet, low in simple carbohydrates, similar to the DIRECT [23] and CENTRAL [13] trials. Both MED and green-MED diets were equally calorie-restricted (men: 1500–1800 kcal/day; women: 1200–1400 kcal/day); ~ 40% of total fat was mainly from polyunsaturated fatty acids (PUFA) and monounsaturated fatty acids (MUFA) and consisted of less than 40 g/day carbohydrates in the first 2 months with increased gradual intake up to 80 g/day. In addition, both MED groups included 28 g/day of walnuts (containing ~ 440 mg polyphenols/day; gallic acid equivalents (GAE), including mostly ellagitannins, ellagic acid, and its derivatives, Phenol-Explorer database). Participants from the green-MED diet were instructed to avoid red and processed meat and were guided to consume 3–4 cups/day of green tea and 100 g of frozen *Wolffia globosa* (Mankai cultivated strain) [26, 27, 47–49] plant cubes (~ 20 g dry Mankai). A specific strain of *Wolffia globosa*, an aquatic plant in the duckweed family, is characterized by high protein content (more than 45% of the dry matter) and the presence of 9 essential and 6 conditional amino acids [48]. The Mankai plant is rich in insoluble fibers, vitamins (including vitamin B12 and folic acid), and minerals (including iron and zinc). We guided the participants to prepare a green Mankai shake with additional ingredients, part of the diet regimen (fruits, walnuts, or vegetables) each evening. The green protein shake was partially substituted for dinner, replacing beef/poultry protein sources (S5). In total, both MED diets had the same calorie restriction. Green tea and Mankai provided an additional daily intake of ~ 800 mg polyphenols [47] [GAE, Phenol-Explorer, and Eurofins laboratory analysis] beyond the polyphenol content in the MED diet. Green tea contains mainly epigallocatechin (EGC), epicatechin gallate (ECG), and epigallocatechin gallate (EGCG) [52]. As previously reported [47], Mankai mostly included the following phenolic metabolites: ellagic acid, benzoic acid, naringenin, luteolin, quercetin, p-coumaric acid, and caffeic acid, analyzed by mass spectrometry-based metabolomics methods from three different laboratories. Participants received green tea, walnuts, and Mankai onsite, free of charge. The participants were instructed to follow their lifestyle intervention for 18 months. The lifestyle interventions included 90-min nutritional and PA sessions in the workplace with multidisciplinary guidance (physicians, clinical dietitians, and fitness instructors). These sessions were held every week during the first month, once a month over the following 5 months, and every other month until the 18th month. All lifestyle educational programs were provided at the same intensity to all three groups. Text messages with relevant information for each assigned intervention group were sent at fixed time intervals to keep the participants motivated. In addition, a website listing all nutritional and PA information needed for the participants was accessible to them according to their intervention group. Most clinical and medical measurements, as well as lifestyle intervention sessions, were conducted onsite. We assessed adherence by self-reported dietary intake and lifestyle habit assessment tool, using validated food frequency questionnaires at baseline and after 6 and 18 months [30], including PA, measured in metabolic equivalent (MET) units. The questionnaire includes 127 food items and 3 portion size pictures for 17 selected food items and a physical activity questionnaire. In addition, the participant’s closed workplace enabled monitoring the freely provided lunch and the presence of an onsite clinic. A detailed description of the provided foods is reported in S5.

### Outcome measures

The abdominal fat depots were assessed at two time points, baseline and 18 months thereafter, using 3-T MRI (Philips Ingenia 3.0T) scans [13]. We quantified abdominal fat using MATLAB-based semiautomatic software [13] and blinded to the intervention group and calculated mean VAT, deep SAT, and superficial SAT along with two axial slices: L5-S1 and L4-L5. Interclass [13] and intraclass reliability were *r* > 0.96 (*p* < 0.001). The full protocol is reported in S6. Anthropometric parameters (i.e., weight and WC) and blood and urine biomarkers were taken at three time points, baseline and after 6 months and 18 months of intervention(S7). Polyphenol compounds were measured in both plasma and urine samples(S7).

### Statistical analysis

The co-primary outcomes of the DIRECT PLUS study were 18-month changes in abdominal fat, the previously published intrahepatic fat (IHF) [28], and obesity. A flow chart of the study is presented in Additional file 1: Fig. S1. For this report, we examined the effect of the green-MED diet on changes in VAT and both deep and superficial SAT over 18 months. We further evaluated the associations between the changes in abdominal adipose tissues with changes in blood biomarkers [13] and consumption of specific foods. Changes in abdominal fat tissues were computed as changes relative to baseline [(time 18 − time0)/time 0 × 100]. Continuous variables are presented as the means (standard deviations). Nominal variables are expressed as numbers and percentages. The Kolmogorov-Smirnov test was used to determine whether variables were normally distributed, and natural log transformations were applied when necessary to achieve normal distributions. Differences in values over time were tested using a paired sample *T*-test or the Wilcoxon test for 18-month changes or three time points using ANOVA for repeated measures. The differences across the groups were tested using ANOVA, the Kruskal-Wallis test, or the chi-square statistic. Correlations were tested using Spearman’s or Pearson’s correlation analysis. The Kendall tau correlation was used to examine the trend of *p*. Multiple comparisons were adjusted using the Tukey post hoc test (for ANOVA) and Bonferroni correction (for Kruskal-Wallis). We used general and generalized linear regression models for adjustments and interaction models (with the specific adjustments detailed in the results). We calculated the cardiovascular risk score using the Systematic Coronary Risk Evaluation (SCORE) [53]. Of the 294 participants, almost all MRI scans at baseline (*n* = 286; 97%) were eligible for abdominal adipose tissue analyses; losses were due to technical reasons. The 18-month primary analyses of abdominal adipose tissues included all 286 participants with intention-to-treat (ITT) analysis [13], imputing for missing observations of 59 participants at follow-up using multiple imputation techniques [54], wherein the following predictors were used in the imputation model: age, sex, baseline weight, and WC at 18 months [13, 28]. We used the most recent values [33] for missing weight and WC data, as was used in the IHF paper as part of the DIRECT PLUS [28]. A sensitivity analysis revealed similar results from the per-protocol analysis, using data from completers only and the ITT analysis (S2). For urine polyphenolic compounds in untargeted analyses, zero values were imputed to the lowest detected, value once log2 transformed. Benjamini-Hochberg correction [55] with a 5% false discovery rate (FDR) was applied to control for multiple comparisons when comparing correlations between change in polyphenolic compounds and VAT (presented in Fig. 3 as urolithin A, the only polyphenolic compound that was statistically significant). Sample size and power calculations are reported in S8. Statistical significance was set at a two-sided *α* = 0.05; analyses were performed using SPSS (version 25.0).

## Supplementary Information

**Additional file 1: S1.** Adherence to the intervention. **S2.** Sensitivity analysis. **S3.** Inclusion and Exclusion criteria. **S4.** Physical activity recommendations protocol. **S5.** Polyphenol-rich foods, provided at no cost to participants. **S6.** Magnetic resonance imaging. **S7.** Clinical parameters, laboratory methodology, and blood and urine polyphenols assessments. **S8.** Sample size and power calculations. **Fig. S1.** DIRECT PLUS flow chart. **Fig. S2.** Heatmap of abdominal adipose depots and metabolic and cardiovascular parameters at baseline. **Fig. S3.** The effect of green Mediterranean diet on 18-month abdominal adipose tissues change, men only (*n*=252). **Fig. S4.** Illustrative MRI image. **Fig. S5.** The association between Mankai consumption and lipid profile change among the green-MED group (DIRECT PLUS). **Fig. S6.** Interaction model of red meat consumption and serum folate change (tertiles) for VAT% dynamics. **Table S1.** Outline of dietary and PA recommendations.

## Data Availability

The majority of results corresponding to the current study are included in the article or uploaded as supplementary material. No further data are available.

## Abbreviations

CVD: Cardiovascular disease
ECG: Epicatechin gallate
EGC: Epigallocatechin
EGCG: Epigallocatechin gallate
FDR: False discovery rate
GAE: Gallic acid equivalents
HDG: Healthy dietary guidelines
IHF: Intrahepatic fat
MED: Mediterranean
MET: Metabolic equivalent
PA: Physical activity
SAT: Subcutaneous adipose tissue
T2D: Type 2 diabetes
VAT: Visceral adipose tissue
WC: Waist circumference
