# Supplementary material for: The effect of high-polyphenol Mediterranean diet on visceral adiposity: the DIRECT PLUS randomized controlled trial
Source: BMC Med. 2022 Sep 30;20:327. doi: 10.1186/s12916-022-02525-8 (PMC9523931; doi:10.1186/s12916-022-02525-8)
Supplement: Supplementary file 1 — Additional file 1: S1. Adherence to the intervention. S2. Sensitivity analysis. S3. Inclusion and Exclusion criteria. S4. Physical activity recommendations protocol. S5. Polyphenol-rich foods, provided at no cost to participants. S6. Magnetic resonance imaging. S7. Clinical parameters, laboratory methodology, and blood and urine polyphenols assessments. S8. Sample size and power calculations. Fig. S1. DIRECT PLUS flow chart. Fig. S2. Heatmap of abdominal adipose depots and metabolic and cardiovascular parameters at baseline. Fig. S3. The effect of green Mediterranean diet on 18-month abdominal adipose tissues change, men only (n=252). Fig. S4. Illustrative MRI image. Fig. S5. The association between Mankai consumption and lipid profile change among the green-MED group (DIRECT PLUS). Fig. S6. Interaction model of red meat consumption and serum folate change (tertiles) for VAT% dynamics. Table S1. Outline of dietary and PA recommendations. [file 12916_2022_2525_MOESM1_ESM.docx]

Online-Only Supplemental Material

[S1: Adherence to the intervention 1](#_Toc108014827)

[S2: Sensitivity analysis 2](#_Toc108014828)

[S3: Inclusion and Exclusion criteria 2](#_Toc108014829)

[S4: Physical activity recommendations protocol 2](#_Toc108014830)

[S5: Polyphenol-rich foods, provided at no cost to participants 2](#_Toc108014831)

[S6: Magnetic resonance imaging 3](#_Toc108014832)

[S7: Clinical parameters, laboratory methodology, and blood and urine polyphenols assessments 3](#_Toc108014833)

[S8: Sample size and power calculations 4](#_Toc108014834)

[Figure S1: DIRECT PLUS flow chart 5](#_Toc108014835)

[Figure S2. Heatmap of abdominal adipose depots and metabolic and cardiovascular parameters at baseline 6](#_Toc108014836)

[Figure S3. The effect of green Mediterranean diet on 18-month abdominal adipose tissues change, men only (n=252) 6](#_Toc108014837)

[Figure S4. Illustrative MRI image 7](#_Toc108014838)

[Figure S5. The association between Mankai consumption and lipid profile change among the green-MED group (DIRECT PLUS) 8](#_Toc108014839)

[Figure S6. Interaction model of Red meat consumption and serum folate change (tertiles) for VAT% dynamics 9](#_Toc108014840)

[Figure Legends 9](#_Toc108014841)

[Table S1: Outline of dietary and PA recommendations 10](#_Toc108014842)

[References 11](#_Toc108014843)

##

## S1: Adherence to the intervention

The participants in the green-MED diet reported an average (weighted mean of both 6- and 18-month reports) green tea consumption of 2.7(1.5)/day (median=2.7 cups/day) and weekly consumption of Mankai of 2.6(1.8)/week (median=2.3 shakes/week). Both MED groups reported a similar intake of walnuts (median amount per day=28g, p=0.91) and a higher frequency walnut intake compared to HDG (p<0.001 HDG vs. MED; p<0.001 HDG vs. green-MED). Furthermore, over 18 months, the MED groups had similar carbohydrate intake reductions (MED:-29.4%(31.9); green-MED:-29.1%(31.7), p=0.79) compared to the HDG group (-14.4%(36.4), p=0.02 vs. green-MED and p=0.01 vs. MED). There were no significant differences in PA intensity levels, measured in MET units, across groups after 18 months of intervention (p=0.85).

## S2: Sensitivity analysis

A per-protocol analysis (data including completers only) revealed results similar to the ITT analysis. The 18-month abdominal adipose depots percent reduction among only the completers divided by group intervention is presented accordingly: VAT (HDG: -5.1%(19.3), MED: -9.1%(21.0), and green-MED: -15.1%(26.8)); deep SAT (HDG: -3.1%(11.9), MED: -7.5%(13.0), and green-MED: -12.0%(16.5)); and superficial SAT (HDG: -3.8%(13.0), MED: -6.9%(14.3), and green-MED: -9.0%(17.4)). The green-MED diet promoted a greater decrease in VAT as compared to other intervention groups (green-MED vs. HDG: p=0.001; green-MED vs. MED: p=0.03). The 18-month reductions in body weight and WC among only the completers were weight loss (HDG: -0.6%(5.2), MED: -3.1%(5.1), and green-MED: -4.4%(6.9)) and WC loss (HDG: -3.9%(5.3), MED: -5.3%(5.0), and green-MED: -6.4%(5.9)). Weight loss and WC reduction after 18 months were similar between the two MED diets (p>0.05 for all) compared to the HDG (weight: HDG vs. MED: p=0.02; HDG vs. green+MED: p<0.001; WC: HDG vs. MED: p=0.24, HDG vs. green+MED: p=0.01).

In analysis among men (Figure S3), which represent the major cohort, differences in VAT% loss between green-MED and other groups remained significant after adjusting for both WC (green-MED vs. MED p=0.007, green-MED vs. HDG p=0.002) or weight change (green-MED vs. MED p=0.011, green-MED vs. HDG p=0.029). This analysis was conducted as a secondary analysis due to the small sample size of women and large diversity in VAT proportion at baseline among women.

## S3: Inclusion and Exclusion criteria

Inclusion criteria: 30+ years of age with abdominal obesity^1^ [waist-circumference(WC): men>102cm, women>88cm] or dyslipidemia^2^ [triglycerides>150mg/dL and high-density-lipoprotein-cholesterol (HDL-c): men≤40mg/dL, women:≤ 50mg/dL]. Exclusion criteria included an inability to partake in physical activity (PA), a serum creatinine level≥2 mg/dL, disturbed liver function, a major illness that might require hospitalization, pregnancy or lactation, presence of active cancer or chemotherapy treatment within the past three years, participation in another trial, current treatment with warfarin (due to the interaction with vitamin K), and implants that would preclude magnetic resonance imaging.

## S4: Physical activity recommendations protocol

The aerobic effort increased gradually, starting with 20 minutes of aerobic training at 65% maximum heart rate, and increased to 45-60 minutes of aerobic training at 80% maximum heart rate. The full workout program included 45-60 minutes of aerobic training 3-4 times/week. Resistance training started with one set of weights corresponding to 60% of the maximum weight, eventually reaching the use of two sets of weights corresponding to 80% of the maximum weight. The resistance training included leg extensions, leg curls, squats, lateral pull-downs, push-ups, shoulder presses, elbow flexions, triceps extensions, and bent-leg sit-ups. The PA recommendations were delivered as part of the 90-minute nutritional and PA sessions in the workplace with multidisciplinary guidance (physicians, clinical dietitians, and fitness instructors). In addition, a website listing all PA information needed for the participants was accessible to them. Assessment of lifestyle habits including PA was performed using validated self-reported food-frequency questionnaires at baseline, after 6- and 18-months^3–5^. The PA was measured in metabolic equivalent (MET) units^6^.

## S5: Polyphenol-rich foods, provided at no cost to participants

Walnuts (groups MED, green-MED): The main polyphenols in walnuts are ellagitannins, ellagic acid, and their derivatives^7^. Walnuts are considered to have a beneficial effect on health maintenance and disease prevention ^8,9^. Additionally, ellagitannins found in nuts were reported to reduce WC, LDL-c, and TG^10^. The nutrition composition of walnuts (28g), taken from the USDA: energy:183 kcal, protein: 4.3g, lipids: 18.3g, carbohydrates: 3.8, fiber: 1.9g, total saturated fatty acids– 1.7g, total monounsaturated fatty acids– 2.5g, total polyunsaturated fatty acids – 13.2g.

Green tea (group green-MED)**:** an unfermented tea produced from the leaves of *Camellia sinensis,* prepared by drying and steaming the leaves, and is a rich source of polyphenols^11^. Most of the polyphenols found in green tea are catechins (the monomer form of flavanols), mainly epigallocatechin (EGC), epicatechin gallate (ECG), and epigallocatechin gallate (EGCG)^12,13^. Short-term (weeks long) intervention studies and meta-analyses have found an association of green tea or its extracts with improvements in cardiometabolic health^14,15^, weight reduction^13^, and cognitive function^16,17^. The participants were required to pour heated water over the green tea bag and steep for 3 minutes.

*Wolffia globosa* duckweed - *Mankai* (group green-MED): A specific strain of *Wolffia globosa,* an aquatic plant in the duckweed family^18–20^, can serve as a plant protein source. In Asian cuisines, *Wolffia globosa* is considered a natural food source or "vegetable meatball"^19^. Nutritionally, *Mankai* is characterized by high protein content (more than 45% of the dry matter) and the presence of 9 essential and 6 conditional amino acids^21^. In addition, it is a good source of omega-3 fatty acids^22^. The Mankai plant is rich in insoluble fibers, vitamins (including vitamin B12)^23^, minerals (including iron and zinc), and polyphenols, including ellagic acid, benzoic acid, naringenin, luteolin, quercetin, p-coumaric acid, and caffeic acid^24,25^. The nutrition composition of Mankai (values for 100gr of frozen cubes Mankai shake (~20g dry): Energy:80 kcal, protein: 9g, lipids: 1.6g, carbohydrates: 7.5, fiber: 1.9g, total saturated fatty acids– 1.7g.

Mankai provides bioavailable essential amino acids^21^ and iron^26^ and has a beneficial effect on postprandial and fasting glycemic control^27^. We guided the participants to prepare a green Mankai shake with additional ingredients, which were also part of the diet regimen (fruits, walnuts, or vegetables) each evening. The green protein shake partially substituted for dinner, replacing beef/poultry protein sources.

## S6: Magnetic resonance imaging

Abdominal fat depots were assessed at baseline and 18-months thereafter using 3-Tesla MRI scans (Ingenia 3.0T, Philips Healthcare, Best, the Netherlands). The scanner utilized a 3D modified DIXON (mDIXON) imaging technique without gaps (2 mm thickness and 2 mm of spacing), fast-low-angle shot (FLASH) sequence with a multi-echo two excitation pulse sequence for phase-sensitive encoding of fat and water signals (TR, 3.6ms; TE1,1.19ms; TE2, 2.3ms; FOV 520×440×80mm; 2×1.4×1mm voxel size). Four images of phantoms were generated: in-phase, out-phase, fat, and water phase^28^. Participants were instructed to hold their breath to avoid motion artifacts when their abdomen was scanned. We quantified abdominal fat using MATLAB-based semiautomatic software and blinded to intervention group. A continuous line over the fascia superficialis was drawn to differentiate deep-SAT from superficial-SAT and calculated mean VAT, deep-SAT, and superficial-SAT along two axial slices: L5-S1 and L4-L5. We quantified fat mass regions as area and relative proportion of each fat subtype (percentage).

## S7: Clinical parameters, laboratory methodology, and blood and urine polyphenols assessments

Clinical parameters:

Measurements were taken at baseline, 6- and after 18-months of intervention. Height was measured to the nearest millimeter using a standard wall-mounted stadiometer. Bodyweight was measured without shoes to the nearest 0.1 kg. WC was measured halfway between the last rib and the iliac crest to the nearest millimeter by standard procedures using an anthropometric measuring tape. Two blood pressure (BP) measurements were recorded after resting using an automatic BP monitor (Accutorr-4, Datascope); the mean of the two was calculated.

Laboratory methodology:

Blood and urine samples were obtained at 8:00 AM after a 12-hour fast. Blood samples were centrifuged, and both blood and urine samples were stored at -80°C. Serum total cholesterol (TC; coefficient-of-variation (CV), 1.3%), HDL-c, low-density-lipoprotein-cholesterol (LDL-c), and TG (CV, 2.1%) were determined enzymatically with a Cobas 8000 automatic analyzer (Roche). Plasma levels of high-sensitivity C-reactive protein (hsCRP) were measured by a Tina-quant® hsCRP assay from Roche. Plasma glucose levels were measured by Roche GLUC3 (hexokinase method). Plasma insulin levels were measured with a Roche Elecsis assay. Serum folate was measured by the electrochemiluminescence immunoassay (ECLIA) as a marker for green leaf consumption^29^. The homeostatic model of insulin resistance (HOMA-IR) was calculated as follows: insulin (µIU/ml)×glucose (mg/dl)/405)^30^. All biochemical analyses were performed at the University of Leipzig, Germany.

Plasma polyphenol metabolites:

The determination of polyphenol metabolites was performed according to the method of Vrhovsek et al^31^ with some modifications. Briefly, a previously developed targeted metabolomics method was performed with an ultra-performance liquid chromatographic system coupled to a tandem mass spectrometry system with electrospray ionization (UHPLC-ESI-MS/MS). Before injection, samples were thawed at 4 °C. Sample preparation was performed using an Ostro™ Pass-through 96-well plate to remove phospholipids and proteins (Waters, Milford, MA, USA). An Ostro™ 96-well plate was fixed on top of a 96-well collection plate. Fifty microliters of plasma were pipetted into the wells, followed by the addition of 1% formic acid in acetonitrile (3:1 solvent/sample). The mixture was then quickly shaken for 5 minutes to promote protein precipitation. Vacuum (15 in. (∼381 mm) Hg) was then applied to the Ostro plate through a vacuum manifold, filtering out the non phospholipid plasma components. This step was repeated twice to ensure protein precipitation. Then, samples were dried under nitrogen and reconstituted in 100 μl of methanol: water (1:1, v/v) containing hippuric acid D5 (1 μg/ml) as an external standard. Samples were finally transferred to LC vials and injected (2 μL) into the UHPLC–MS/MS system. All solvents were kept at 4 °C before use, and all procedures were carried out in a cold room with a relatively short extraction time (10 minutes). Quality control (QC) samples were also prepared prior to analysis by pooling a small fraction of all the individual analyzed samples. Data processing was performed using Waters MassLynx 4.1 (Waters, Milford, CT, USA) and TargetLynx software (Waters, Milford, CT, USA). Details of the liquid chromatography and mass spectrometry are described in Vrhovsek et al^31^ and Gasperotti et al^32^.

Urine polyphenol metabolites:

Acetonitrile (ACN) and water of LC-MS analytical grade were purchased from J.T. Baker (part of Fisher Scientific). Formic acid was purchased from Honeywell (Charlotte, NC, USA), and β-glucuronidase (EC: 3.2.1.31)-type HP-2 from *H. pomatia* was obtained from Sigma Aldrich (St. Louis, MO, USA).

Urine samples were thawed at RT for 5-10 min. For enzymatic deconjugation, 15 µl β-glucuronidase was added to 50 µl urine and incubated for 2 h at 37 °C. Samples were then extracted twice by adding 600 µl ethyl acetate and shaking for 5 minutes at 1100 rpm. The organic phases were combined and centrifuged (15 min, 3000 rpm) to remove impurities. Subsequently, the supernatant was dried in a SpeedVac™ vacuum concentrator (Eppendorf) and stored.

Prior to LC-MS/MS measurement, the samples were resuspended in water with 1% ACN and 0.1% formic acid, and 10 µl was injected into an HPLC-QToF instrument from Agilent Technologies (6540 UHD Accurate-Mass Q-TOF LC/MS instrument). Metabolites were loaded on a C18-precolumn (Acquity BEH C18 1.8 µm, 2.1 x 50 mm) and separated on a C18 column (Acquity UPLC HSS T3 1.8 µm, 2.1 x 100 mm) at a flow rate of 0.3 mL/min with the following gradient of running solvent A (0.1% formic acid in water) and running solvent B (0.1% formic acid in acetonitrile): 0-5 min 1% B, 5.1-20 min 1%-100% B, 20.1-25 min 1% B. All samples were acquired in positive and negative ionization mode. The QToF was set up in centroid mode and in screening mode, allowing the detection of ions with a mass-to-charge ratio between 60 and 1000. After each full scan, the 5 most intense ions (threshold 200 counts) were fragmented.

Raw files (.d) were imported into Progenesis QI® software (v.2.1, Waters Corporation). Samples in different ionization modes were processed separately. The workflow included isotope and adduct fusion and chromatogram alignment in the t_R_ direction based on a reference chromatogram. Next, peak picking was applied using default sensitivity settings. A database search was performed using ChemSpider as an identification method with the urine human metabolome database^33^ and Phenol explorer^34^ as input selection. The precursor and fragment mass tolerances were set to 15 ppm and 10 ppm, respectively. Only precursor peaks with corresponding fragment spectra were retained. Normalized peak areas and possible identifications were exported.

The exported possible feature identifications were filtered using in-house written R scripts^35^. Briefly, feature identifications were filtered for a Progenesis score of at least 40. Then, for each feature, only the top-scoring identification and those with a score less than 5 lower than the top score were kept. Finally, the resulting filtered data were further analyzed using a second in-house written R-script to extract possible polyphenolic compounds in the identification list. Only identifications where the polyphenolic compound was the top-scoring hit or shared top-scoring hit were annotated as a polyphenolic compound.

## S8: Sample size and power calculations

The sample size and power calculation for the visceral fat changes were based on findings from our previous CENTRAL trial^36^ that resulted in a significant reduction in VAT (-47.3(36.6) visceral fat reduction in the MED diet vs. -32.9(33.5) in the reference low-fat diet; 14.4 difference; power= 90%, alpha= 5%). The calculation for the sample size needed for this trial suggested 83 participants in each intervention group and ultimately recruited a number of 98 participants per group (~90 in each group with a valid MRI scan). The power calculation was 81.56%. Sample size and power calculations were performed using Winpepi software, version 11.6.

## Figure S1: DIRECT PLUS flow chart


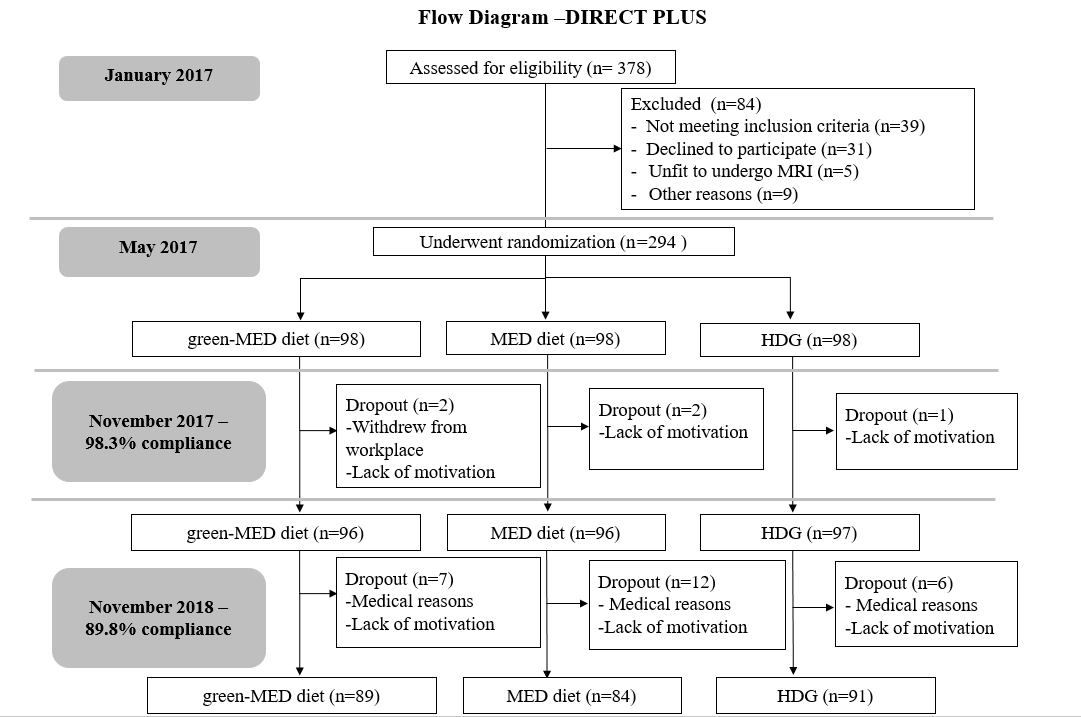


## Figure S2. Heatmap of abdominal adipose depots and metabolic and cardiovascular parameters at baseline

## Figure S3. The effect of green Mediterranean diet on 18-month abdominal adipose tissues change, men only (n=252)

## Figure S4. Illustrative MRI image


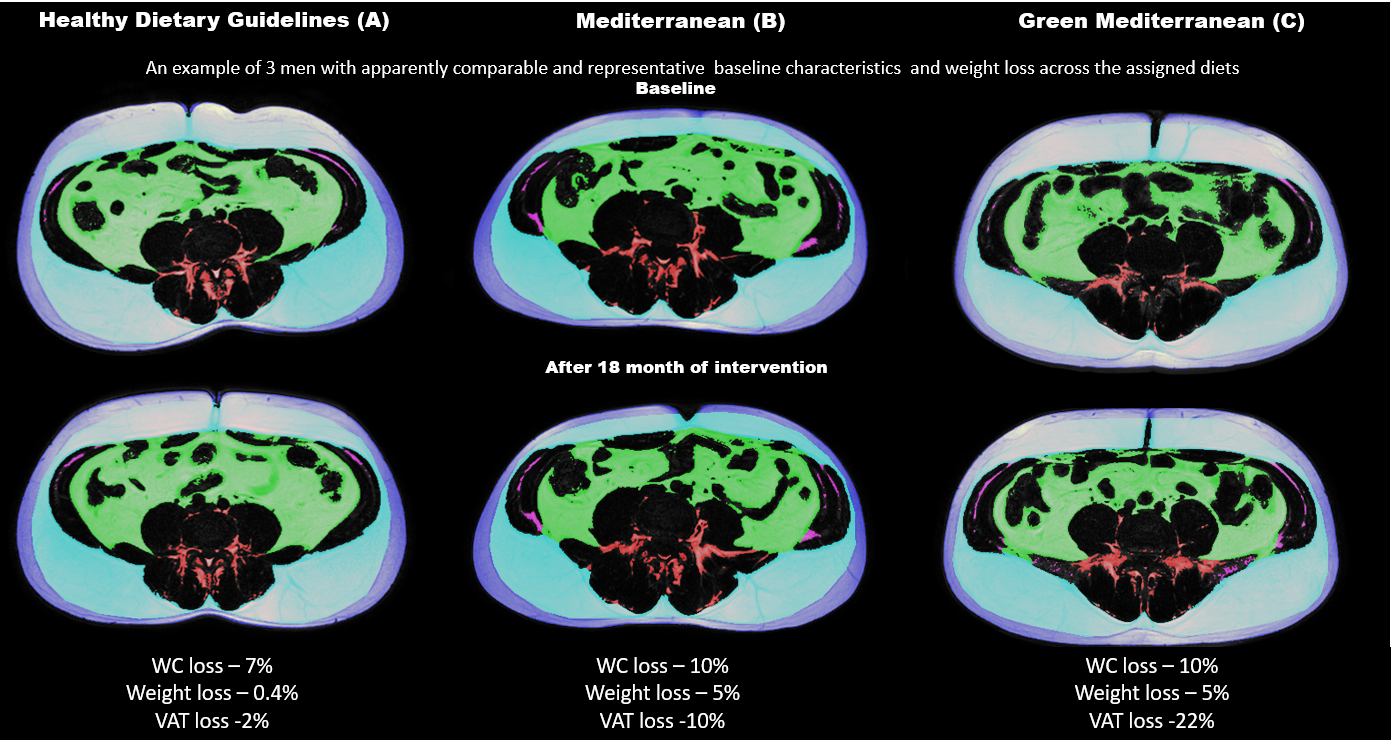


## Figure S5. The association between Mankai consumption and lipid profile change among the green-MED group (DIRECT PLUS)

##

## Figure S6. Interaction model of Red meat consumption and serum folate change (tertiles) for VAT% dynamics

# Figure Legends

Figure S1. DIRECT PLUS flow chart

Figure S2. Heatmap of abdominal adipose depots and metabolic and cardiovascular parameters at baseline. Baseline correlations between the three abdominal adipose depots, clinical parameters and cardiovascular risk score. VAT area at baseline was more strongly correlated with WC (r=0.54, p<0.001) than with bodyweight (r=0.38, p<0.001). Greater VAT was associated with higher cardiovascular risk score, systolic and diastolic blood pressure, triglycerides, glucose, HOMA-IR, and IL-6 and lower HDL-c levels (all p<0.05). In contrast, greater superficial-SAT was associated with lower cardiovascular risk score, systolic blood pressure and triglycerides and higher HDL-c levels (all p<0.05). At the extremes of the color gradient, red represents the strongest positive correlation (r = +1), and blue represents the strongest inverse correlation (r = -1); white or no color represents no correlation (r = 0). Cardiovascular risk score include the Systematic Coronary Risk Evaluation (SCORE); HDL, High-density lipoprotein; HOMA-IR, homeostatic model of insulin resistance; IL-6, interleukin 6; LDL, low-density lipoprotein; SAT, subcutaneous adipose tissue; VAT, visceral adipose tissue. * denotes significant correlation at 0.05 level.

Figure S3. 18-month changes in abdominal adipose tissues (mean(SE)) between intervention groups among men population (intention-to-treat analysis, n=252). After 18 months of intervention, all groups reduced all three abdominal adipose tissues significantly. Significant differences in VAT% change between the green-MED group and MED, as well as HDG groups, were observed adjusted for age, waist circumference change (green-MED vs. MED p=0.007, green-MED vs. HDG p=0.002) as presented or weight change (green-MED vs. MED p=0.011, green-MED vs. HDG p=0.029). MED, Mediterranean; SAT, subcutaneous adipose tissue. * denotes significant within-group change vs. baseline at 0.05 level. ** states significant differences between groups at 0.05 level.

Figure S4. Illustrative MRI image: a comparison of three male participants with similar baseline characteristics (age, WC, weight and VAT) who were randomized to either HDG (participant A: Man, Age- 47 y, baseline measurements: WC –112cm, VAT – (131cm2) 27%, weight – 92kg), MED group (participant B:Man, Age- 51 y, baseline measurements: WC – 109cm, VAT – (113cm2) 30%, weight- 92kg) or green-MED group (participant C:Man, Age- 46 y, baseline measurements: WC – 116cm, VAT – (124cm2) 25%, weight- 103kg). Despite the similar WC loss across the MED groups, they exhibited different reductions in VAT. WC, waist circumference; VAT, visceral adipose tissue.

Figure S5. 18-month changes in lipid biomarkers and cardiovascular risk score (mean(SE)) between Mankai consumption groups (green MED diet only), adjusted for age and weight-loss. Cardiovascular risk score include the Systematic Coronary Risk Evaluation (SCORE). Mankai consumption categories (18 months): Low/non: <=1/week, Medium:2-3/week, High:3>/week;* denotes significant within-group change vs. baseline at 0.05 level. ** states significant differences between groups at 0.05 level.

Figure S6. Interaction model for red meat consumption change at time 18 and 18-month serum folate change (tertiles) for 18m VAT% change adjusted for age and sex.*p of interaction; Serum folate tertiles (of 18m change in serum folate change): T1≤-0.41; T2= -0.40 to 1.46; T3=1.47+.

## Table S1: Outline of dietary and PA recommendations

| **Green-MED** | | **MED** | **HDG** |  |
| --- | --- | --- | --- | --- |
| 18-months group sessions in the workplace, weekly for the first month and monthly thereafter  18 months free gym membership  18 months of PA educational sessions  45-60 minutes of aerobic training + resistance training, 3-4 times/week | | | | Lifestyle group sessions, including PA |
| Limit dietary cholesterol, trans-fat, saturated fat, sugars, and salt and increase intake of vegetables | | | | General dietary guidance |
| 1500-1800 kcal/day for men, 1200-1400 kcal/day for women | | | Guidelines for a healthy MED diet with no specific recipes or calorie restriction | Energy, kcal/day |
| ~40% mainly PUFA and MUFA | | |  | Total fat, % of daily consumption |
| Less than 40 gr/day in the first 2 months with increased gradual intake for up to 80 gr/day | | |  | Carbohydrates, gr/day |
| Less red and processed meats. Reduced poultry intake | | |  | Specific recommendations |
| +1240 mg/day  [source: provided walnuts (28 g/day), green tea (3-4 cups/day), Wolffia globosa duckweed (Mankai) shake (100 g frozen cubes)] | +440 mg/day  [source: provided  walnuts (28g/day] | |  | Polyphenols, mg/day |

# References

1. Lean, M. E., Han, T. S. Morrison, C. E. Waist circumference as a measure for indicating need for weight management. BMJ 311, 158–161 (1995).

2. Expert Panel on Detection, Evaluation, and Treatment of High Blood Cholesterol in Adults. Executive Summary of The Third Report of The National Cholesterol Education Program (NCEP) Expert Panel on Detection, Evaluation, And Treatment of High Blood. Cholest Adults (Adult Treat Panel III) JAMA 2001;2852486–2497pmid1136870

3. Shai I, Shahar DR, Vardi H, Fraser D. Selection of food items for inclusion in a newly developed food-frequency questionnaire. Public health nutrition 2004; 7(6): 745-9.

4. Shai I, Rosner BA, Shahar DR, et al. Dietary evaluation and attenuation of relative risk: multiple comparisons between blood and urinary biomarkers, food frequency, and 24-hour recall questionnaires: the DEARR study. J Nutr 2005 ;135(3)573-9

5. Gepner Y, Golan R, Harman-Boehm I, et al. Effects of Initiating Moderate Alcohol Intake on Cardiometabolic Risk in Adults With Type 2 Diabetes: A 2-Year Randomized, Controlled Trial. Ann Intern Med 2015 163(8):569.

6. Ainsworth BE, Haskell WL, Whitt MC, et al. Compendium of physical activities: an update of activity codes and MET intensities. Med Sci Sports Exerc 2000;32:S498-504.

7. Regueiro J, Sánchez-González C, Vallverdú-Queralt A, Simal-Gándara J, Lamuela-Raventós R, Izquierdo-Pulido M. Comprehensive identification of walnut polyphenols by liquid chromatography coupled to linear ion trap–Orbitrap mass spectrometry. Food Chem. 2014;152:340-8

8. Sánchez-González C, Ciudad C, Noé V, Izquierdo-Pulido M. Health benefits of walnut polyphenols: An exploration beyond their lipid profile. Crit Rev Food Sci Nutr. 2015.

9. Kris-Etherton PM. Walnuts decrease risk of cardiovascular disease: A summary of efficacy and biologic mechanisms. J Nutr. 2014 Apr;144(4 Suppl):547S-54S.

10. García-Conesa M, Chambers K, Combet E, et al. Meta-analysis of the effects of foods and derived products containing ellagitannins and anthocyanins on cardiometabolic biomarkers: Analysis of factors influencing variability of the individual responses. Int J Mol Sci 2018;19(3)694

11. Pérez-Jiménez J, Neveu V, Vos F, Scalbert A. Identification of the 100 richest dietary sources of polyphenols: An application of the phenol-explorer database. Eur J Clin Nutr [Internet]. 2010;64:S112-20.

12. Manach C, Scalbert A, Morand C, Remesy C, Jimenez L. Polyphenols: Food sources and bioavailability. Am J Clin Nutr 2004 May;79(5)727-47

13. Huang J, Wang Y, Xie Z, Zhou Y, Zhang Y, Wan X. The anti-obesity effects of green tea in human intervention and basic molecular studies. Eur J Clin Nutr [Internet]. 2014;68(10):1075-87.

14. Zheng XX, Xu YL, Li SH, Liu XX, Hui R, Huang XH. Green tea intake lowers fasting serum total and LDL cholesterol in adults: A meta-analysis of 14 randomized controlled trials. Am J Clin Nutr [Internet]. 2011 Aug;94(2):601-10.

15. Hooper L, Kroon PA, Rimm EB, et al. Flavonoids, flavonoid-rich foods, and cardiovascular risk: A meta-analysis of randomized controlled trials. Am J Clin Nutr [Internet]. 2008 Jul;88(1):38-50.

16. Park S, Jung I, Lee WK, et al. A combination of green tea extract and l-theanine improves memory and attention in subjects with mild cognitive impairment: A double-blind placebo-controlled study. J Med food 2011;14(4)334-43

17. Wightman EL, Haskell CF, Forster JS, Veasey RC, Kennedy DO. Epigallocatechin gallate, cerebral blood flow parameters, cognitive performance and mood in healthy humans: A double‐blind, placebo‐controlled, crossover investigation. Hum Psychopharmacol Clin E. 2012;27(2):177-86

18. Landolt, E. Lemnaceae Gray Duckweed Family. Flora North Am. 22, (2014).

19. Bhanthumnavin K, McGarry MG. Wolffia arrhiza as a possible source of inexpensive protein. Nature 232, 495 (1971).

20. Kawamata Y, Shibui Y, Takumi A, Seki T, Shimada T, Hashimoto M, Inoue N, Kobayashi H NT. Genotoxicity and repeated-dose toxicity evaluation of dried Wolffia globosa Mankai. Toxicol Rep2020 Sep 14;71233-1241

21. Kaplan A, Zelicha H, Tsaban G, et al. Protein bioavailability of Wolffia globosa duckweed, a novel aquatic plant, – A randomized controlled trial. Clin Nutr [Internet] 2018;Available from: https://doi.org/10.1016/j.clnu.2018.12.009

22. Yan Y, Candreva J, Shi H, Ernst E, Martienssen R, Schwender J SJ. Survey of the total fatty acid and triacylglycerol composition and content of 30 duckweed species and cloning of a Δ6-desaturase responsible for the production of γ-linolenic and stearidonic acids in lemna gibba. BMC plant Biol [Internet] 2013;13(1)201

23. Sela, I. Yaskolka Meir, A. Brandis, A. et al. Wolffia globosa–Mankai Plant-Based Protein Contains Bioactive Vitamin B12 and Is Well Absorbed in Humans. Nutrients 2020, 12, 3067.

24. Daduang J, Daduang S, Hongsprabhas P, Boonsiri P. High phenolics and antioxidants of some tropical vegetables related to antibacterial and anticancer activities. African Journal of Pharmacy and Pharmacology. 2011;5(5):608-15.

25. Edelman M, Colt M. Nutrient Value of Leaf vs . Seed. Front Chem 2016;4(July):2–6.

26. Yaskolka Meir A, Tsaban G, Zelicha H, et al. A green Mediterranean diet, low in meat and supplemented with duckweed, does not impair iron homeostasis in obese, dyslipidemic adults or rats. J Nutr 2018;1–8.

27. Zelicha H, Kaplan A, Meir AY, et al. The effect of wolffia globosa mankai, a green aquatic plant, on postprandial glycemic response: A randomized crossover controlled trial. Diabetes Care 2019;42(7):1162–9.

28. Thomas EL, Fitzpatrick JA, Malik SJ, Taylor-Robinson SD, Bell JD. Whole body fat: content and distribution. Prog Nucl Magn Reson Spectrosc. 2013;73:56-80. doi: 10.1016/j.pnmrs.2013.04.001.

29. Moll R, Davis B. Iron, vitamin B12 and folate. Medicine 2017;45:198-203.

30. Matthews DR, Hosker JP, Rudenski AS, Naylor BA, Treacher DF TR. Homeostasis model assessment: insulin resistance and betacell function from fasting plasma glucose and insulin concentrations in man. Diabetol 1985;28(7)412-9

31. Vrhovsek U, Masuero D, Gasperotti M, Franceschi P, Caputi L, Viola R, Mattivi F. A versatile targeted metabolomics method for the rapid quantification of multiple classes of phenolics in fruits and beverages. J Agric Food Chem [Internet]. 2012;60(36):8831.

32. Gasperotti M, Masuero D, Guella G, Mattivi F, Vrhovsek U. Development of a targeted method for twenty-three metabolites related to polyphenol gut microbial metabolism in biological samples, using SPE and UHPLC–ESI-MS/MS. Talanta [Internet]. 2014;128:221-3.

33. Wishart DS, Feunang YD, Marcu A, et al. HMDB 4.0: the human metabolome database for 2018. Nucleic Acids Res 2018;46:D608-d17.

34. Rothwell JA, Perez-Jimenez J, Neveu V, et al. Phenol-Explorer 3.0: a major update of the Phenol-Explorer database to incorporate data on the effects of food processing on polyphenol content. Database 2013;2013.

35. Ihaka R, Gentleman R. R: A Language for Data Analysis and Graphics. Journal of Computational and Graphical Statistics 1996;5:299-314.

36. Gepner Y, Shelef I, Schwarzfuchs D, et al. Effect of distinct lifestyle interventions on mobilization of fat storage pools CENTRAL magnetic resonance imaging randomized controlled trial. Circulation 2018;137(11):1143–57.
